# The specific shapes of capillaries are associated with worse prognosis in patients with invasive breast cancer

**DOI:** 10.1111/pin.13442

**Published:** 2024-05-31

**Authors:** Hnin‐Wint‐Wint Swe, Masayoshi Fujisawa, Toshiaki Ohara, Yu Komatsubara, Teizo Yoshimura, Tadahiko Shien, Akihiro Matsukawa

**Affiliations:** ^1^ Department of Pathology and Experimental Medicine, Graduate School of Medicine, Dentistry and Pharmaceutical Sciences Okayama University Okayama Japan; ^2^ Department of Breast and Endocrine Surgery Okayama University Hospital Okayama Japan

**Keywords:** angiogenesis, blood vessels, breast cancer, CD31 antigen, immunohistochemistry, microvessel density, survival analysis

## Abstract

Angiogenesis is considered essential for tumor progression; however, whether histological counting of blood vessel numbers, expressed as microvessel density (MVD), can be a prognostic factor in breast cancer remains controversial. It has been suggested that the specific morphology of blood vessels such as glomeruloid microvascular proliferation (GMP) is associated with clinical parameters. Here, we aimed to clarify the significance of MVD with revised immunohistochemistry and to identify new blood vessel shapes that predict prognosis in breast cancer. Four hundred and eleven primary breast cancer specimens were collected, and the sections were immunohistochemically stained with CD31 (single staining) and CD31 and Collagen IV (double staining). The prognosis of patients was examined based on the MVD value, and the presence of GMP and other blood vessels with other specific shapes. As a result, high MVD value and the presence of GMP were not associated with worse prognosis. By contrast, patients with deep‐curved capillaries surrounding tumor cell nests (C‐shaped) or excessively branched capillaries near tumor cell nests showed a significantly poor prognosis. The presence of these capillaries was also correlated with clinicopathological parameters such as Ki‐67 index. Thus, the morphology of capillaries rather than MVD can be a better indicator of tumor aggressiveness.

## Graphical abstract

Blood vessel density and shapes were histologically analyzed in 411 invasive breast cancer cases. Microvessel density was not a reliable indicator for survival even with revised immunohistochemistry. By contrast, patients with irregularly shaped blood vessels, such as deep‐curved capillaries surrounding tumor cell nests (C‐shaped) or excessively branched capillaries near tumor cell nests, showed a significantly poor prognosis and correlations with clinicopathological parameters such as Ki‐67 index.

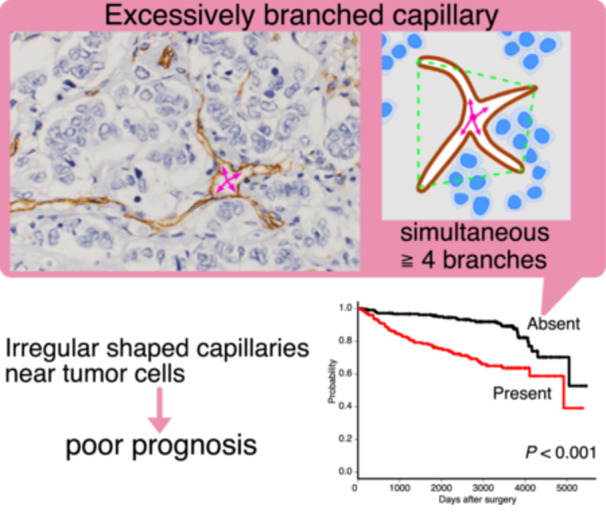

**Abbreviations**
CSS: cancer‐specific survival
DFS: disease‐free survival
GMP: glomeruloid microvascular proliferation
MVD: microvessel density
OS: overall survival

## INTRODUCTION

Breast cancer is one of the most common causes of cancer death among women. Several prognostic factors have already been identified in breast cancer. These include tumor stage, histological grade, molecular subtypes, Ki‐67 index, lymph node metastasis, and lymphatic vessel invasion.^1^, ^2^, ^3^, ^4^, ^5^, ^6^, ^7^

Angiogenesis is essential for tumor progression and histological evaluation of angiogenesis is assumed to be a prognostic indicator in solid tumors.^8^ Microvessel density (MVD), the number of blood vessels per given microscopic area, has been widely used to estimate tumor angiogenesis. However, the counting method and values of MVD are not consistent among studies. MVD can be influenced by several factors, such as field area, whether counting is performed manually or automatically, and whether it is expressed as a counted number or Chalkley score.^9^, ^10^, ^11^ In addition, a variety of endothelial markers are used for the identification of blood vessels. The relationship between MVD and patient prognosis in invasive breast cancer remains controversial: some studies reported that high MVD was associated with worse survival, consistent with the theoretical assumption.^12^, ^13^, ^14^ Others failed to demonstrate this relationship or even showed an inverse relationship: high MVD had better survival.^15^, ^16^, ^17^

The choice of endothelial markers appears to have a major impact on the results, as the staining characteristics vary between markers. For example, factor VIII is a relatively insensitive marker and it was not expressed in immature endothelial cells in tumors.^18^ CD31 and CD34 are more sensitive markers and the counts obtained by using them were approximately 30% higher than using factor VIII.^19^ Plasma cells and fibroblasts can react with CD31 and CD34, respectively, interfering with blood vessel identification.^18^

In addition to the number of blood vessels, the morphological features of blood vessels have been introduced as prognostic indicators. Cavitary structure type blood vessels have been found to be associated with different features of breast cancer,^20^ and glomeruloid microvascular proliferation (GMP), originally introduced as a glioblastoma marker, has also been reported to be a marker of aggressive behavior in breast cancer.^21^, ^22^ There may be other vascular morphologies that predict cancer prognosis.

In this study, we have developed an immunohistochemical double staining method (CD31 and collagen IV) that overcomes the above problems because all endothelial cells (labeled with CD31) are surrounded by vascular basement membrane (labeled with collagen IV). Using this method, we investigated the relationship between MVD and prognosis in breast cancer. Next, we evaluated shapes of blood vessels which potentially predict prognosis and found that distinctive structures seen in a subset of our cases, such as deeply curved capillaries surrounding tumor cell nests (C‐shaped) or excessively branched capillaries, were associated with poor prognosis.

## MATERIALS AND METHODS

### Case selection

Four hundred and eleven cases of invasive breast carcinoma without preoperative chemotherapy were collected from the Pathology database of Okayama University Hospital. Pathological data such as estrogen receptor (ER), progesterone receptor (PR), HER2, and Ki‐67 status were also retrieved from routine reports. The patients’ operation dates were from 2008 to 2012 and clinical follow‐up data were up to October 2023. Lymph nodes had been spared in 14 patients for clinical reasons. Four patients had synchronous bilateral tumors. These tumors were counted independently, but the survival was analyzed as one person: only the tumor with the first event was taken into account. The detailed clinicopathological characteristics are described in Supporting Information S3: Table S1. Although individual written consent was not obtained, our study plan and the patients’ and families’ right of opting out were disclosed on our website, and cases unmet with refusal were enrolled in the study. This study was approved by the institutional review boards of Okayama University on October 24, 2017 (1710‐042).

### Tissue staining by immunohistochemistry

One representative block with the most abundant tumor tissue was used for each case. Serial tissue sections of 4 μm thickness were prepared. H&E, D2‐40, CD31, CD31, and collagen IV double staining were performed by the conventional immunohistochemistry method. Elastin and collagen IV double staining was performed as described previously.^23^ Complete information on the antibodies and the staining procedures can be found in Supporting Information S3: Tables S2–S4.

### Histological analysis

Histological grade and fibrotic focus were determined using prevalent systems.^24^, ^25^, ^26^ Lymphatic vessel invasion and blood vessel invasion were analyzed as described previously.^23^ MVD, GMP, C‐shaped, and excessively branched capillaries were investigated for each case. In the invasive tumor areas, blood vessels were identified by brown‐stained structures with or without lumen using CD31 single staining. Structures with red‐colored insides (CD31) and brown‐colored outsides (collagen IV) were regarded as blood vessels using double staining.

MVD was assessed in the hotspot, the area that contained the highest number of capillaries in each tumor, identified under low power magnification. The sum of the number of blood vessels in 10 consecutive fields counted under high power magnification (×40 objective lens) was adopted as MVD.^27^ The median value was used to determine the cutoff value (median = 65, range = 16–168 for single staining and median = 87, range = 21–307 for double staining).

GMP and other specific structures were recorded throughout the whole section as either absent or present. GMP was identified as renal glomerulus‐like aggregates of CD31‐positive (single staining) and CD31 and collagen IV‐positive (double staining) endothelial cells. Although lumen formation was not necessary, the number of endothelial cells in aggregates of endothelial cells should be 15–100.^28^ C‐shaped and excessively branched capillaries were identified according to the criteria described in the Results section. Three individuals (M.F., H.‐W.‐W. S., and Y. K.) observed the slides independently and discussed the findings until they reached an agreement.

### Morphometrical analysis

To quantitatively characterize the shape of abnormal vessels, the areas containing excessively branched capillaries and surrounding regularly shaped blood vessels were scanned. Digital images were taken by a digital camera (DP73, Olympus) attached to a microscope (BX43, Olympus) with ×20 objective lens (UPlanSAPo ×20, Olympus) from CD31 single‐stained slide. The image was binarized to blood vessel and background areas using a color‐picking tool of an image processing software (Winroof, Mitani Corporation) followed by minor manual correction. The median filter with radius 2 pixel was used to remove noise. Minimal Feret diameter, perimeter, and solidity of each blood vessel was calculated using the particle analysis function of Image J software.

### Three‐dimensional (3D) image analysis based on thick sections

Cases with C‐shaped and excessively branched capillaries identified in thin sections were classified into two‐dimensional (2D)‐positive and 2D‐negative groups, respectively. Ten cases were chosen at random from each group and sections with 50 µm thickness were prepared. Immunofluorescence staining was done by a free‐floating method according to Supporting Information S3: Table S5.

Under low magnification, four to seven foci rich in both tumor cells and blood vessels were chosen for each case and their full thickness was scanned with a ×40 silicone immersion objective lens (Olympus, confocal laser scanning biological microscope, FV1000). 3D images and animations were created using Imaris AI microscopy imaging software (version 10.1). The number of C‐shaped and excessively branched capillaries was counted, and the mean value per visual field between 2D‐positive and 2D‐negative groups was compared.

### Statistical analysis

The correlation analyses between conventional clinicopathological parameters and the blood vessel parameters were examined by Fisher's exact test or *t*‐test. For survival analyses, cancer‐specific survival (CSS), disease‐free survival (DFS), and overall survival (OS) were examined using the Kaplan–Meier curves. The log‐rank test was used to compare survival differences between the groups. DFS was adopted as a representative indicator of survival, and CSS and OS were further analyzed if the difference in DFS was significant. Multivariate analysis (multiple logistic regression analysis and Cox's proportional hazards analysis) was applied only to compare the prognostic effects of parameters. *p* Values smaller than 0.05 were defined as statistically significant. Statistical analysis was performed using EZR software.^29^

## RESULTS

### MVD analysis by single and double staining

**Figure 1:**
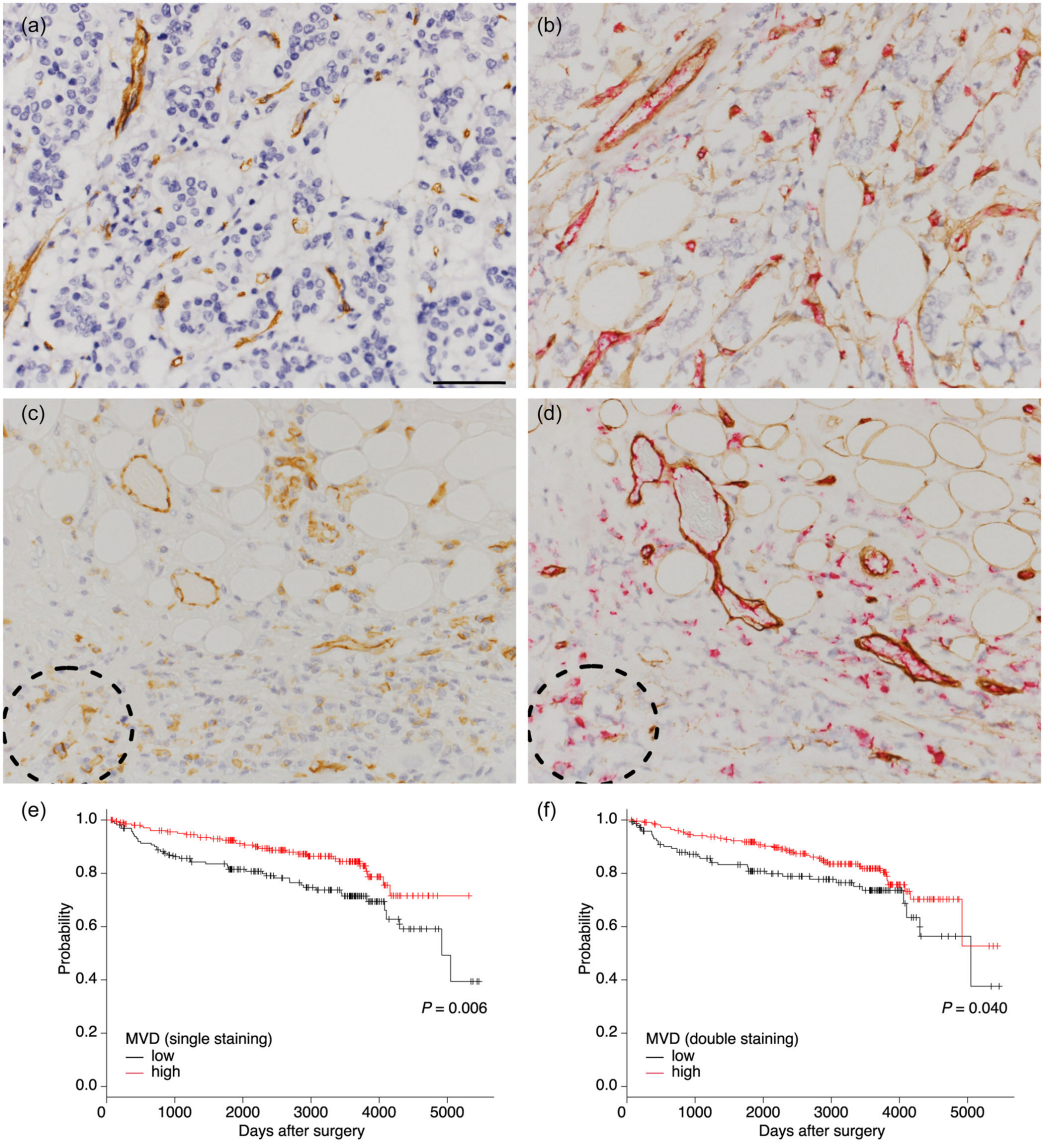
Assessment of microvessel density (MVD) by single and double staining and its association with prognosis. (a, b) A case with high MVD. Single staining (a) and double staining of the matched area (b). (c, d) A case with low MVD. Single staining (c) and double staining of the matched area (d). Blood vessels were stained brown by single CD31 staining and stained red inside (CD31) and brown outside (Collagen IV) by double staining. CD31‐positive cells in the dotted circles do not have a basement membrane, indicating they are not endothelial cells. (e, f) Kaplan–Meier survival curves for disease‐free survival with single staining (e) or double staining (f). The scale bar indicates 50 µm.

We first analyzed MVD using both CD31 single staining and CD31 and collagen IV double staining slides. CD31 single staining often had undesirable signals on plasma cells and macrophages, whereas blood vessels were identified as red (CD31, endothelial cells) and brown (collagen IV, basement membrane) stained structures by double staining and were easily distinguished from inflammatory cells (Figure 1a–d).

We observed that the group with higher MVD showed better survival by both staining methods (Figure 1e,f). Significant correlations between most of the clinicopathological parameters and MVD were not found by either single or double staining. The details of our findings are shown in Supporting Information S3: Table S6.

### GMP

Next, we focused on GMP, which is a known morphological type of blood vessels in cancer tissues. We found that 10.9% and 21.9% of cases had GMP by single and double staining, respectively. By both staining methods, GMP was associated with several clinicopathological parameters, such as histological grade, estrogen receptors, progesterone receptors, Ki‐67 index, and subtypes (Supporting Information S3: Table S7). The cases with GMP showed a trend towards worse survival, although it was not statistically significant (Supporting Information S1: Figure S1).

### C‐shaped capillaries

**Figure 2:**
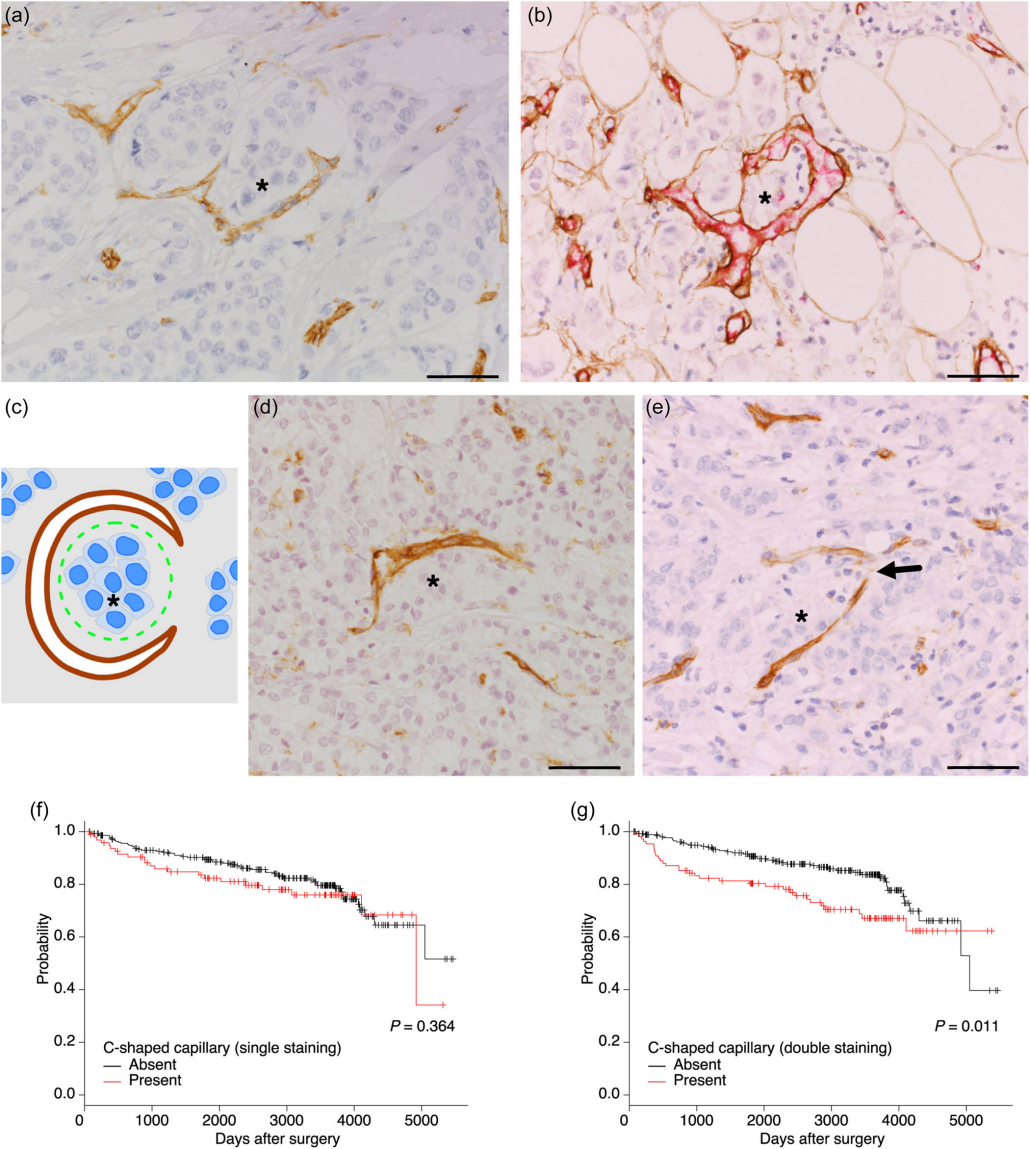
C‐shaped capillaries and their association with prognosis. (a, b) C‐shaped capillaries that encircle tumor cells (asterisks). Blood vessels were stained brown by single CD31 stain (a) and stained red inside (CD31) and brown outside (Collagen IV) by double staining (b). (c) An illustration showing the criterion for determination of C‐shaped capillaries. A capillary (brown) continuously encircles at least one tumor cell or cell nest (asterisk) more than 50% (180°). (d, e) Capillaries which were not regarded as C‐shaped (CD31). The capillary's encirclement of tumor cell nests (asterisk) was less than 50% (d) or was interrupted indicated by arrow (e). (f, g) Kaplan–Meier survival curves of patients with and without C‐shaped capillaries for disease‐free survival by single staining (d) and double staining (e). Scale bars indicate 50 µm.

We then focused on previously undescribed structures found in invasive tumor areas of some subsets of cases, one being C‐shaped capillary. C‐shaped capillary was a deeply curved capillary that encircled more than half of a tumor cell nest and tended to embrace the tumor cells (Figure 2a,b). For consistent identification of this structure, a strict criterion was applied: C‐shaped capillary continuously encircled at least one tumor cell or cell nest by more than 50% (180°) (Figure 2c). If the encirclement of a blood vessel was less than half or not continuous, this structure was not regarded as a C‐shaped capillary (Figure 2d,e). By CD31 single staining, C‐shaped capillaries were brown‐stained structures surrounding tumor cells (Figure 2a). By CD31 and collagen IV double staining, they are red (inside) and brown (outside) double‐stained structures encircling tumor cells (Figure 2b).

The detection rate of this vessel type was 24.8% by single staining and 27.4% by double staining. By Kaplan–Meier analysis, cases with C‐shaped capillaries showed worse survival than those without C‐shaped capillaries when double staining was used for detection (Figure 2f,g). The presence of C‐shaped capillaries showed significant correlations with most clinicopathological parameters, including tumor size, histological grade, Ki‐67 index, and blood vessel invasion regardless of which stain was used. However, C‐shaped capillaries detected by single staining were not associated with lymph node metastasis and lymphatic vessel invasion (Table 1).

**Table 1:** C‐shaped capillaries and clinicopathological factors.

	**C‐shaped capillary (single staining)**			**C‐shaped capillary (double staining)**		
	Absent	Present		Absent	Present	
Parameter	*n* (%)	*n* (%)	*p* Value	*n* (%)	*n* (%)	*p* Value
Total	309 (75.2)	102 (24.8)		298 (72.5)	113 (27.5)	
Tumor size			<0.001*			<0.001*
≤2 cm	239 (77.3)	59 (57.8)		231 (77.5)	67 (59.3)	
>2 cm	70 (22.6)	43 (42.2)		67 (22.5)	46 (40.7)	
Lymph node metastasis			0.608			0.018*
Negative	210 (69.8)	70 (72.9)		215 (73.9)	65 (61.3)	
Positive	91 (30.2)	26 (27.1)		76 (26.1)	41 (38.7)	
Histological grade			<0.001*			<0.001*
Grade 1–2	230 (74.4)	38 (37.3)		223 (74.8)	45 (39.8)	
Grade 3	79 (25.6)	64 (62.7)		75 (25.2)	68 (60.2)	
Estrogen receptor			<0.001*			0.001*
Negative	51 (16.5)	42 (41.2)		55 (18.5)	38 (33.6)	
Positive	258 (83.5)	60 (58.8)		243 (81.5)	75 (66.4)	
Progesterone receptor			<0.001*			0.001*
Negative	77 (24.9)	51 (50%)		78 (26.2)	50 (44.2)	
Positive	232 (75.1)	51 (50.0)		220 (73.8)	63 (55.8)	
HER2 overexpression			0.016*			0.062
Negative	271 (87.7)	79 (77.5)		260 (87.2)	90 (79.6)	
Positive	38 (12.3)	23 (22.5)		38 (12.8)	23 (20.4)	
Ki‐67 index						
≤20%	198 (64.1)	33 (32.4)	<0.001*	190 (63.8)	41 (36.3)	<0.001*
>20%	111 (35.9)	69 (67.6)		108 (36.2)	72 (63.7)	
Subtype			<0.001*			0.009*
ER+ and HER2−	239 (77.3)	52 (51.0)		224 (75.2)	67 (59.3)	
ER+ and HER2+	19 (6.1)	8 (7.8)		19 (6.4)	8 (7.1)	
ER− and HER2+	19 (6.1)	15 (14.7)		19 (6.4)	15 (13.3)	
ER− and HER2−	32 (10.4)	27 (26.5)		36 (12.1)	23 (20.4)	
Lymphatic vessel invasion			0.200			<0.001*
Negative	230 (74.4)	69 (67.6)		233 (78.2)	66 (58.4)	
Positive	79 (25.6)	33 (32.4)		65 (21.8)	47 (41.6)	
Blood vessel invasion			<0.001*			<0.001*
Negative	183 (59.2)	35 (34.3)		184 (61.7)	34 (30.1)	
Positive	126 (40.8)	67 (65.7)		114 (38.3)	79 (69.9)	
Fibrotic focus			<0.001*			<0.001*
Negative	235 (76.1)	46 (45.1)		222 (74.5)	59 (52.2)	
Positive	74 (23.9)	56 (54.9)		76 (25.5)	54 (47.8)	

* *p *< 0.05.

### Excessively branched capillaries

**Figure 3:**
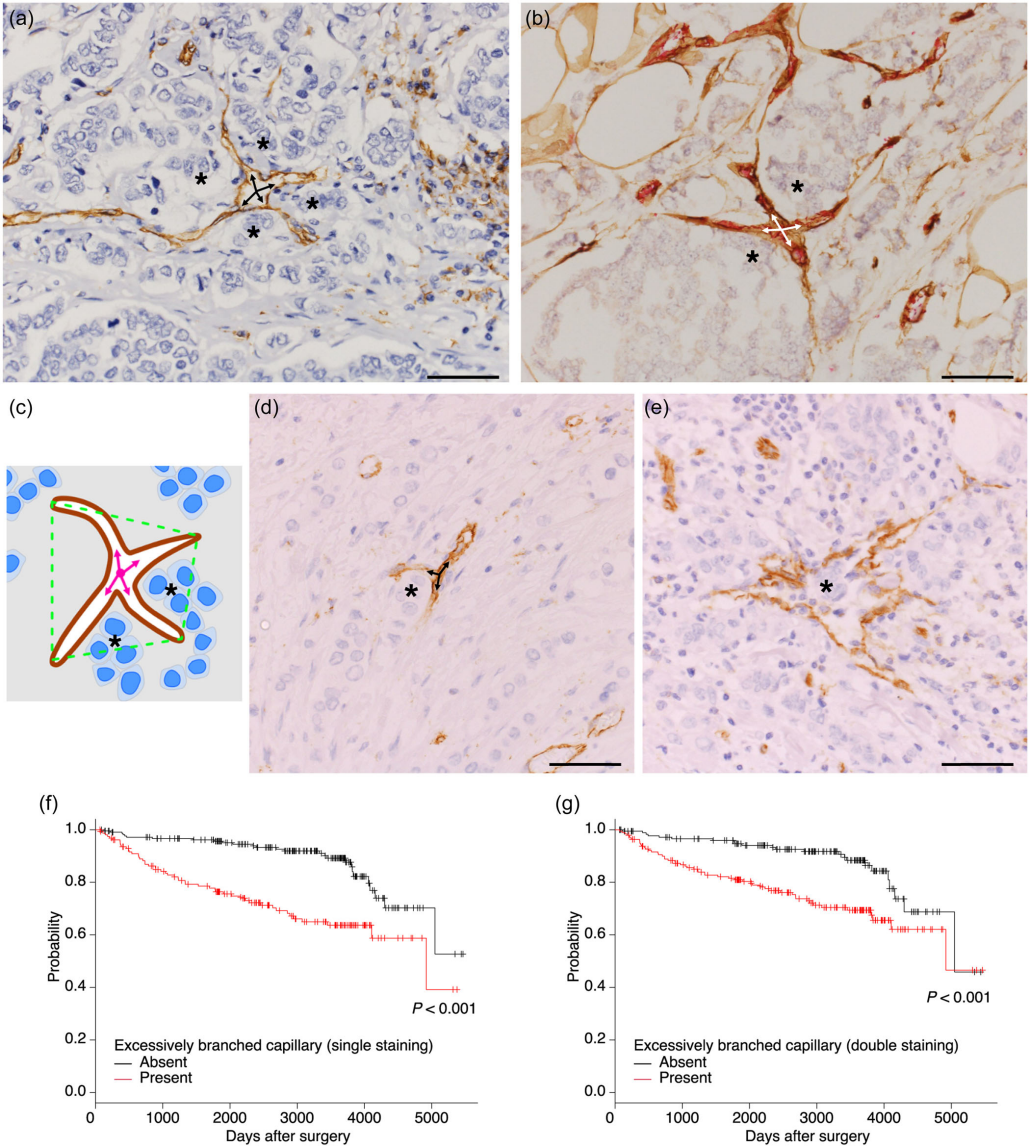
Excessively branched capillaries and their association with prognosis. (a, b) Excessively branched capillaries having four branches (arrows) and tumor cells nearby (asterisks). Blood vessels were stained brown by single CD31 staining (a) and stained red inside (CD31) and brown outside (Collagen IV) by double staining (b). (c) An illustration showing the criteria for determination of excessively branched capillaries. A blood vessel (brown) has at least four immediate branches (arrows) and tumor cells in the vicinity of branches (within the polygon shown in green dotted line) found in invasive tumor areas. (d, e) Capillaries which were not regarded as excessively branched (CD31). A capillary with only three branches indicated by arrows (d). Vessels which had tumor cells (asterisk) within the lumen were almost always lymphatic vessels (lymphatic vessel invasion of tumor cells) (e). (f, g) Kaplan–Meier survival curves of patients with and without excessively branched capillaries for disease‐free survival with single staining (d) and double staining (e). Scale bars indicate 50 µm.

Excessively branched capillaries were the other structures found in some tumor tissues. These were defined as blood vessels with at least four immediate branches accompanied by tumor cells nearby (within the polygon whose apexes were the tips of the branches) which were found in invasive tumor areas (Figure 3a [single staining], b [double staining], and c [illustration]). Vessels with only three branches were not regarded as excessively branched capillaries (Figure 3d). As lymphatic vessels sometimes took similar shapes, they were excluded by comparison with D2‐40 immunostaining (Figure 3e).

Excessively branched capillaries were found in almost 50% of cases using both single and double staining in our study. Survival analysis showed that patients with excessively branched capillaries had a significantly worse prognosis regardless of which staining methodology was used (Figure 3f,g). Moreover, the presence of excessively branched capillaries was correlated with most of the conventional clinicopathological parameters as well as with the presence of C‐shaped capillaries (Table 2). Multivariate models composed of some established prognostic factors and specific shapes of capillaries showed that the presence of excessively branched capillaries, not C‐shaped capillaries, was an independent predictor for DFS, CSS, and OS (Table 3).

**Table 2:** Excessively branched capillaries and clinicopathological factors.

	**Excessively branched capillary (single staining)**			**Excessively branched capillary (double staining)**		
	Absent	Present		Absent	Present	
Parameter	*n* (%)	*n* (%)	*p* Value	*n* (%)	*n* (%)	*p* Value
Total	246 (59.9)	165 (40.1)		208 (50.6)	203 (49.4)	
Tumor size			<0.001*			<0.001*
≤2 cm	209 (85.0)	89 (53.9)		173 (83.2)	125 (61.6)	
>2 cm	37 (15.0)	76 (46.1)		35 (16.8)	78 (38.4)	
Lymph node metastasis			<0.001*			<0.001*
Negative	185 (77.1)	95 (60.5)		161 (79.3)	119 (61.3)	
Positive	55 (22.9)	62 (39.5)		42 (20.7)	75 (38.7)	
Histological grade			<0.001*			<0.001*
Grade 1–2	197 (80.1)	71 (43.0)		170 (81.7)	98 (48.3)	
Grade 3	49 (19.9)	94 (57.0)		38 (18.3)	105 (51.7)	
Estrogen receptor			0.003*			0.001*
Negative	43 (17.5)	50 (30.3)		31 (14.9)	62 (30.5)	
Positive	203 (82.5)	115 (69.7)		177 (85.1)	141 (69.5)	
Progesterone receptor			0.001*			0.002*
Negative	61 (24.8)	67 (40.6)		50 (24.0)	78 (38.4)	
Positive	185 (75.2)	98 (59.4)		158 (76.0)	125 (61.6)	
HER2 overexpression			0.674			<0.001*
Negative	211 (85.8)	139 (84.2)		191 (91.8)	159 (78.3)	
Positive	35 (14.2)	26 (15.8)		17 (8.2)	44 (21.7)	
Ki‐67 index			<0.001*			<0.001*
≤20%	166 (67.5)	65 (39.4)		147 (70.7)	84 (41.4)	
>20%	80 (32.5)	100 (60.6)		61 (29.3)	119 (58.6)	
Subtype			0.009*			<0.001*
ER+ and HER2−	187 (76.0)	104 (63.0)		169 (81.2)	122 (60.1)	
ER+ and HER2+	16 (6.5)	11 (6.7)		8 (3.8)	19 (9.4)	
ER− and HER2+	19 (7.7)	15 (9.1)		9 (4.3)	25 (12.3)	
ER− and HER2−	24 (9.8)	35 (21.2)		22 (10.6)	37 (18.2)	
Lymphatic vessel invasion			<0.001*			<0.001*
Negative	201 (81.7)	98 (59.4)		177 (85.1)	122 (60.1)	
Positive	45 (18.3)	67 (40.6)		31 (14.9)	81 (39.9)	
Blood vessel invasion			<0.001*			<0.001*
Negative	165 (67.1)	53 (32.1)		147 (70.7)	71 (35.0)	
Positive	81 (32.9)	112 (67.9)		61 (29.3)	132 (65.0)	
Fibrotic focus			<0.001*			<0.001*
Negative	198 (80.5)	83 (50.3)		173 (83.2)	108 (53.2)	
Positive	48 (19.5)	82 (49.7)		35 (16.8)	95 (46.8)	
C‐shaped capillary			<0.001*			<0.001*
Negative	230 (93.5)	79 (47.9)		194 (93.3)	104 (51.2)	
Positive	16 (6.5)	86 (52.1)		14 (6.7)	99 (48.8)	

* *p* < 0.05.

**Table 3:** Multivariate analysis with common clinicopathological parameters, C‐shaped, and excessively branched capillaries.

	Disease‐free survival		Cancer‐specific survival		Overall survival	
Explanatory variable	HR (95% CI)	*p* Value	HR (95% CI)	*p* Value	HR (95% CI)	*p* Value
Tumor size (>2 cm vs. ≤2 cm)	1.085 (0.644–1.826)	0.759	1.119 (0.608–2.062)	0.717	0.991 (0.542–1.813)	0.978
Lymph node metastasis (positive vs. negative)	1.329 (0.812– 2.176)	0.258	1.515 (0.844–2.719)	0.164	1.711 (0.966–3.030)	0.066
Estrogen receptor (positive vs. negative)	1.918 (0.997– 3.688)	0.051	2.005 (0.931–4.314)	0.075	1.474 (0.723–3.007)	0.286
HER2 overexpression (positive vs. negative)	1.196 (0.601– 2.378)	0.610	1.021 (0.439–2.378)	0.961	0.945 (0.430–2.082)	0.890
Ki‐67 index (>20% vs. ≤20%)	1.928 (1.128–3.296)	0.016*	2.169 (1.132–4.155)	0.020*	1.757 (0.938–3.292)	0.078
C‐shaped capillaries^a^ (present vs. absent)	0.960 (0.562–1.639)	0.880	1.352 (0.722–2.531)	0.346	0.923 (0.508–1.675)	0.791
Excessively branched capillaries^b^ (present vs. absent)	2.795 (1.559– 5.012)	<0.001*	3.577 (1.674–7.642)	<0.001*	3.198 (1.620–6.313)	<0.001*

Abbreviations: CI, confidence interval; HR, hazard ratio.

^a^ Results by CD31 and collagen Ⅳ double staining.

^b^ Results by CD31 single staining.

* *p* < 0.05.

Then, survival was analyzed by biomarker‐defined subtypes. The presence of excessively branched capillaries was strongly associated with worse prognosis in ER‐positive and HER2‐negative patients, whereas the associations with other subtypes showed either borderline significance (ER‐positive and HER2‐positive patients and ER‐negative and HER2‐positive patients) or none (ER‐negative and HER2‐negative patients) for DFS. The cases with excessively branched capillaries showed a worse prognosis in ER‐positive and HER2‐negative group also for CSS and OS (Supporting Information S1: Figures S2–S4).

To further characterize the shape of excessively branched capillaries, their morphometric parameters such as minimal Feret diameter, perimeter, and solidity were compared with surrounding regularly shaped blood vessels within the digital image field. In 165 cases with excessively branched capillaries, 229 blood vessels were found to be excessively branched capillaries and 8819 were regularly shaped capillaries. Excessively branched capillaries had significantly larger minimal Feret diameter and perimeter than surrounding blood vessels indicating their size is larger. Moreover, these capillaries showed a lower solidity value, indicating their shape is more complex. All biomarker‐defined subtypes shared this tendency (Supporting Information S1: Figure S5).

### 3D analysis based on thick sections

**Figure 4:**
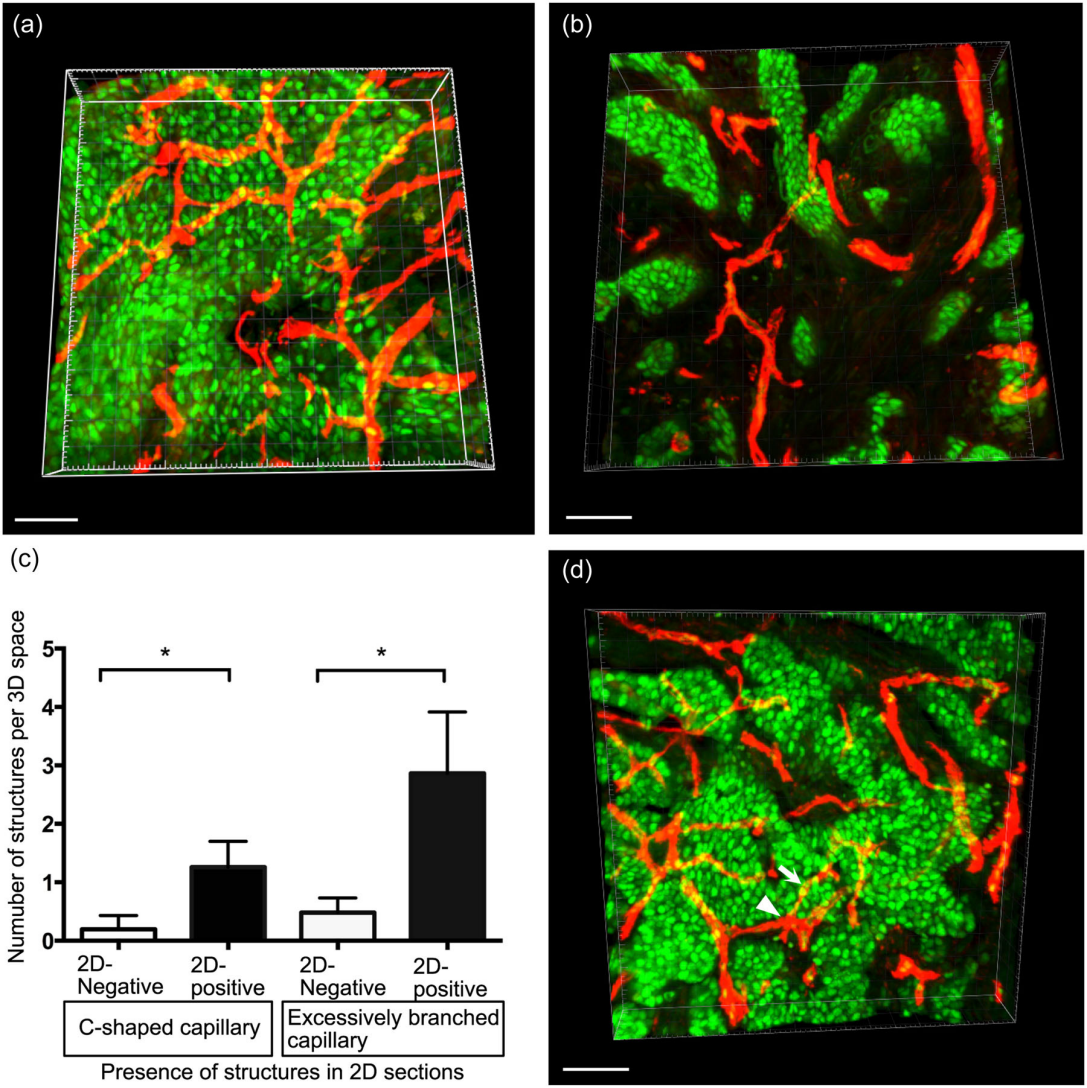
Three‐dimensional analysis of C‐shaped and excessively branched capillaries. (a, b) Three‐dimensional view of thick sections from cases with (a) or without (b) C‐shaped and excessively branched capillaries in thin sections. (c) The number of C‐shaped and excessively branched capillaries in thick sections (*p < 0.001). (d) Colocalization of C‐shaped (thin arrow) and excessively branched capillaries (arrowhead). Red is blood vessels (CD31) and green is tumor cells (GATA3). Scale bars indicate 50 µm.

To know whether the complex structures seen in thin sections reflected the original tissue per se, 3D analysis was performed using a confocal microscope. Since this analysis consumed a significant amount of paraffin blocks, 10 cases each were randomly selected from cases with C‐shaped and excessively branched capillaries (2D‐positive group) and cases without them (2D‐negative group), and 50‐μm‐thick sections were stained. 3D images were generated, and C‐shaped and excessively branched capillaries were identified using the same criteria as in the thin sections (Figure 4a,b).

When tissues in thick sections were three‐dimensionally examined, C‐shaped and excessively branched capillaries were found more frequently than in thin sections and occasionally even in 2D‐negative cases. In thick sections, the number of these capillaries was also found to be significantly higher in 2D‐positive cases than in 2D‐negative cases (Figure 4c). Moreover, nearly 80% (53 out of 67) of the C‐shaped capillaries shared or were close to branches of the excessively branched capillaries, suggesting that these two features were structurally related to each other (Figure 4d). Animations generated from the 3D images are shown in Supporting Information S2: Figure S6.

## DISCUSSION

Cancer cells require angiogenesis for further growth, and researchers in breast cancer and other types of cancer have been interested in whether the number of blood vessels assessed by MVD can predict patient prognosis. In invasive breast cancer, there have been many reports on the correlation between MVD and clinical outcomes; however, the results have not been consistent.

Blood vessels for the estimation of MVD have been highlighted using various endothelial markers, such as CD31 and CD34. The expression of endothelial cell markers varies depending on the type of organ and type of blood vessel, and none of these markers are specific or sensitive to endothelial cells.^30^ The double staining used in this study was helpful in distinguishing endothelial cells from other cells with an undesirable CD31 signal, such as plasma cells and macrophages. Examination using this double staining showed that, contrary to our expectations, high MVD cases had a slightly better prognosis than low MVD cases. Our results suggest that the significance of MVD as a prognostic indicator of invasive breast cancer is limited, regardless of the stain used.

The other aspect of analyzing angiogenesis is the shape of blood vessels. It was also reported different forms of tumor vascularization and their respective implications in liver metastasis of colorectal carcinoma.^31^ Thus, the analysis of blood vessel shapes could be a clue to determining a prognostic factor of breast cancer. One of the shapes of interest is GMP, which was originally introduced for the analysis of glioblastoma and the presence of GMP predicted an impaired prognosis in melanoma.^32^, ^33^, ^34^ In breast cancer, GMP was reported to be associated with features of aggressive characteristics such as estrogen receptor negativity, p53 positivity and resistance to chemotherapy.^22^, ^35^ Moreover, there is an association of GMP with BRCA1 mutations and higher nuclear grade, both of which appear to increase tumor responsiveness to chemotherapy.^31^, ^36^ In our study, GMP was not associated with survival by either single or double staining. GMP tended to be located within the stroma away from tumor cell nests and not in direct contact. Therefore, we focused on specific shapes of capillaries which seemed to be influenced by tumor cells.

C‐shaped capillaries were found in at least 20% of cases using single or double staining, and their presence showed a strong association with established clinicopathological parameters such as histological grade, hormone receptor positivity, and Ki‐67 index. Compared to CD31 single staining, double staining is a more sensitive and better indicator of aggressive behavior. Excessively branched capillary is another morphology that indicates complexity. Normally, blood vessels grow from a main trunk into two branches like hierarchical structures, so they appear as three‐branched tubes on tissue sections.^37^ Simultaneously, four‐branched blood vessels may be a type of abnormal angiogenesis as tumor vessels are often unorganized.^38^ In contrast to C‐shaped capillaries, excessively branched capillaries are strongly correlated with prognosis in both single and double staining. Although the presence of C‐shaped capillaries was strongly associated with that of excessively branched capillaries, their positional relationship in conventional thin sections was unknown. The 3D analysis showed that C‐shaped capillaries were often located in the vicinity of excessively branched capillaries, suggesting they may be different aspects of similar phenomena.

Because excessively branched capillary is recorded as absent or present in the whole section, its detection rate is supposed to depend on the size of the tumor area. Indeed, correlation analysis showed that excessively branched capillaries were found more frequently in large tumors than in small tumors. However, our multivariable model, including tumor size as an explanatory parameter, showed that excessively branched capillary was an independent prognostic factor. Moreover, our 3D analysis, whose observation size was equal throughout the cases, reproduced the detection rate of excessively branched capillaries in thin sections: cases with excessively branched capillaries in thin section (2D‐positive cases) had more numerous these capillaries than cases without them in thin section (2D‐negative cases). Thus, excessively branched capillaries seem to reflect a distinctive character of tumor aggressiveness independent of tumor size.

Among the subtypes of breast cancer, the presence of excessively branched capillaries showed the strongest association with the prognosis of ER‐positive and HER2‐negative subtype. This subtype tends to have regular‐shaped vessels, that is, the least frequency of irregular blood vessels (C‐shaped and excessively branched capillaries) among four subtypes (Tables 1 and 2). In this subtype, there is considerable heterogeneity in patient prognosis and treatment benefit and the treatment strategy for patients with this subtype is not as straightforward as for other subtypes.^39^ Thus, in addition to multigene expression assays that reflect the phenotypes of the tumor cells themselves, excessively branched capillaries may be a new indicator of tumor aggressiveness that reflects the tumor microenvironment. Since excessively branched capillaries are easily accessible using single CD31 staining that is available to most laboratories, this may be applicable to daily practice.

Previous studies have shown that some morphometric parameters of blood vessels are prognostic. Mikalsen et al. showed that larger blood vessel size, indicated by perimeter and more complex shape, indicated by solidity, were associated with poor survival.^40^ Milosevic et al. also showed that larger vessel size, indicated by minimal Feret diameter, was associated with poor prognosis only in ER‐positive breast cancer patients.^41^ Our results are consistent with these observations because excessively branched capillaries were scored larger and more complex‐shaped than regularly shaped capillaries when these parameters were adopted. The previous studies focus on the general morphology of all blood vessels whereas our study focuses on finding the specific shape of abnormal blood vessels among all blood vessels. Although these different approaches share similar results, it is unclear which is more essential for understanding the pathogenesis. Since these studies are based on morphology, the mechanism of the formation of abnormal capillaries and the reason why it is related to prognosis should be clarified in further studies.

In conclusion, MVD does not appear a reliable indicator for survival even with a sensitive and specific staining method (double staining of CD31 and collagen IV). Instead, specific shapes of capillaries, such as C‐shaped and excessively branched capillaries, were associated with worse prognosis. Identification of such specific capillaries can be an additional indicator of tumor aggressiveness, especially for ER‐positive and HER2‐negative patients.

## AUTHOR CONTRIBUTIONS

All authors contributed to the study conceptualization and design. Hnin‐Wint‐Wint Swe and Masayoshi Fujisawa established the methodology. Masayoshi Fujisawa and Tadahiko Shien collected the materials, including clinical information. Hnin‐Wint‐Wint Swe, Masayoshi Fujisawa, and Yu Komatsubara performed the final analysis including staining and histological analysis. Toshiaki Ohara, Akihiro Matsukawa, and Teizo Yoshimura supervised the study. Hnin‐Wint‐Wint Swe wrote the original draft and all authors reviewed and edited the previous versions of the manuscript. All authors read and approved the final manuscript.

## CONFLICT OF INTEREST STATEMENT

None declared.

## ETHICS STATEMENT

This study was approved by the institutional review boards of Okayama University on October 24, 2017 (1710‐042). Although individual written consent was not obtained, our study plan and the patients’ and families’ right of opting out were disclosed on our website, and cases unmet with refusal were enrolled in the study. This consent procedure was approved by the institutional review boards of Okayama University on October 24, 2017 (1710‐042).

## Supporting information

Supporting information.

Supporting information.

Supporting information.

## Data Availability

The data sets generated during the current study are not publicly available due to institutional policy but are available from the corresponding author on reasonable request.
