# Supplementary material for: The specific shapes of capillaries are associated with worse prognosis in patients with invasive breast cancer
Source: Pathol Int. 2024 May 31;74(7):394–407. doi: 10.1111/pin.13442 (PMC11551825; doi:10.1111/pin.13442)
Supplement: Supplementary file 1 — Supporting information. [file PIN-74-394-s001.pdf]

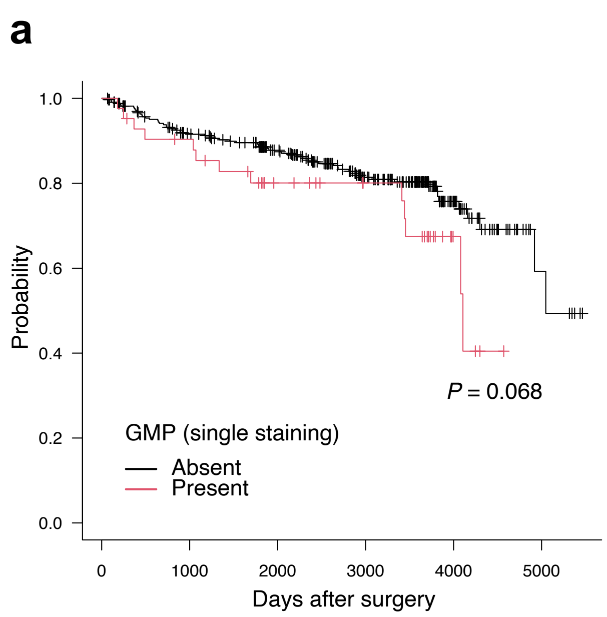

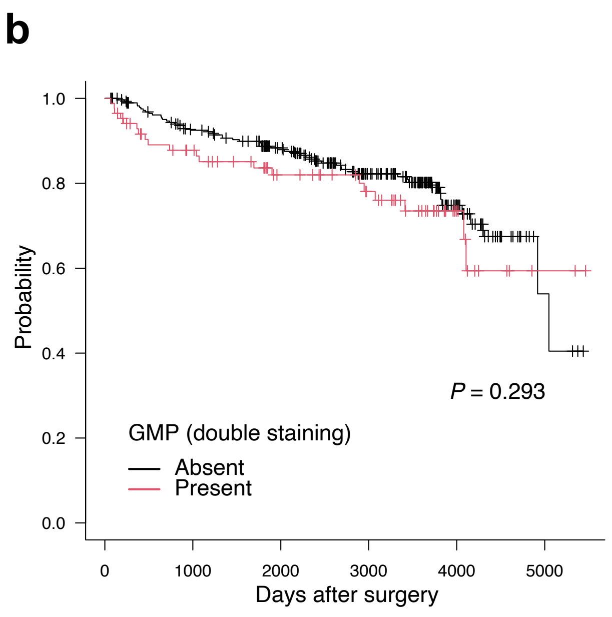

Supplementary Fig S1 Kaplan Meier survival curves of patients with and without glomeruloid microvascular proliferation (GMP) for disease free survival
a:single staining, **b**:double staining

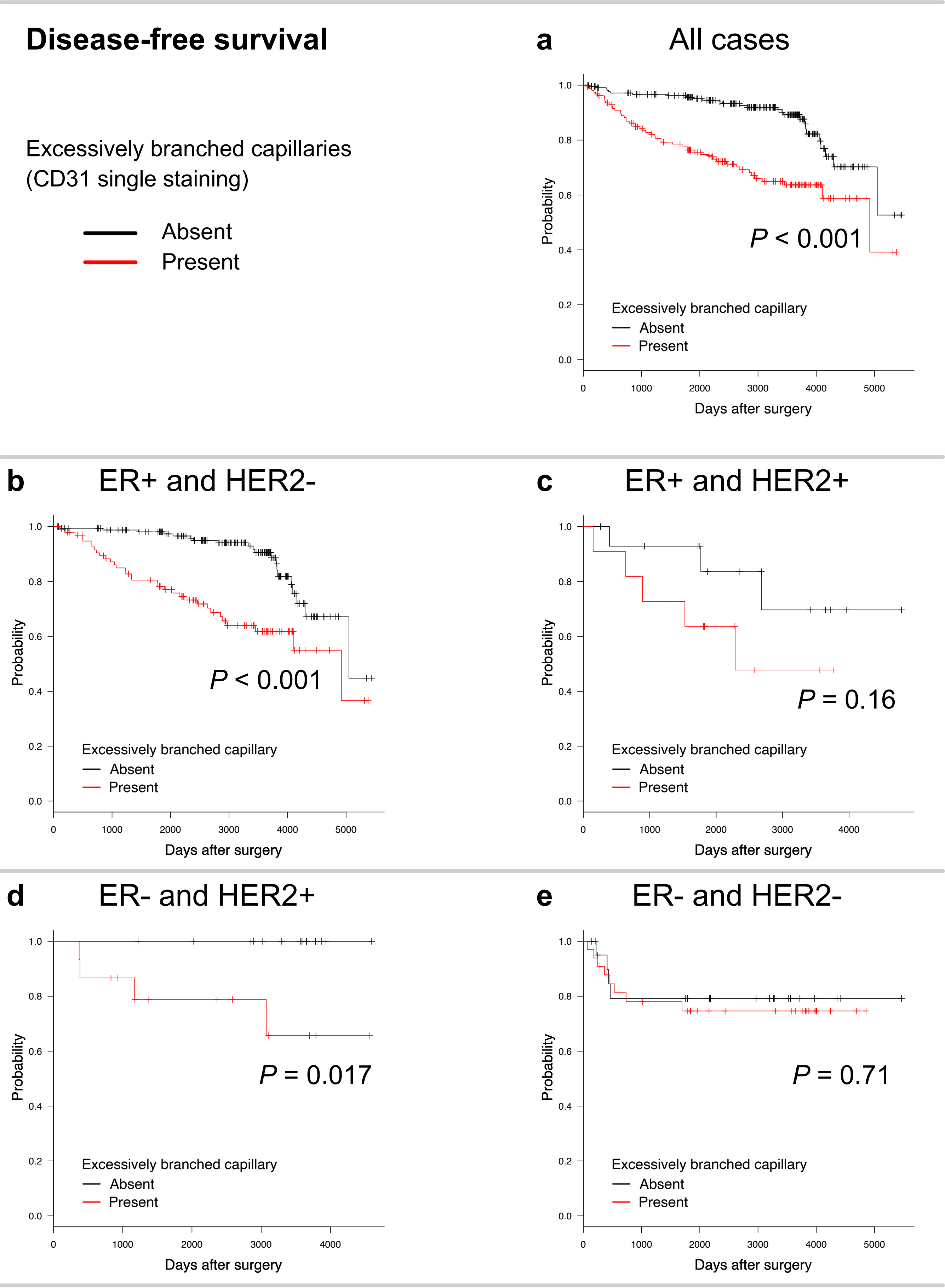

Supplementary Fig S2 Kaplan Meier survival curves of patients with and without excessively branched capillaries for disease-free survival

a: All cases (n = 376)
b: ER-positive and HER2-negative group (n = 263)
c: ER-positive and HER2-positive group (n = 26)
d: ER-negative and HER2-positve group (n = 32)
e: ER-negative and HER2-negative group (n = 55)

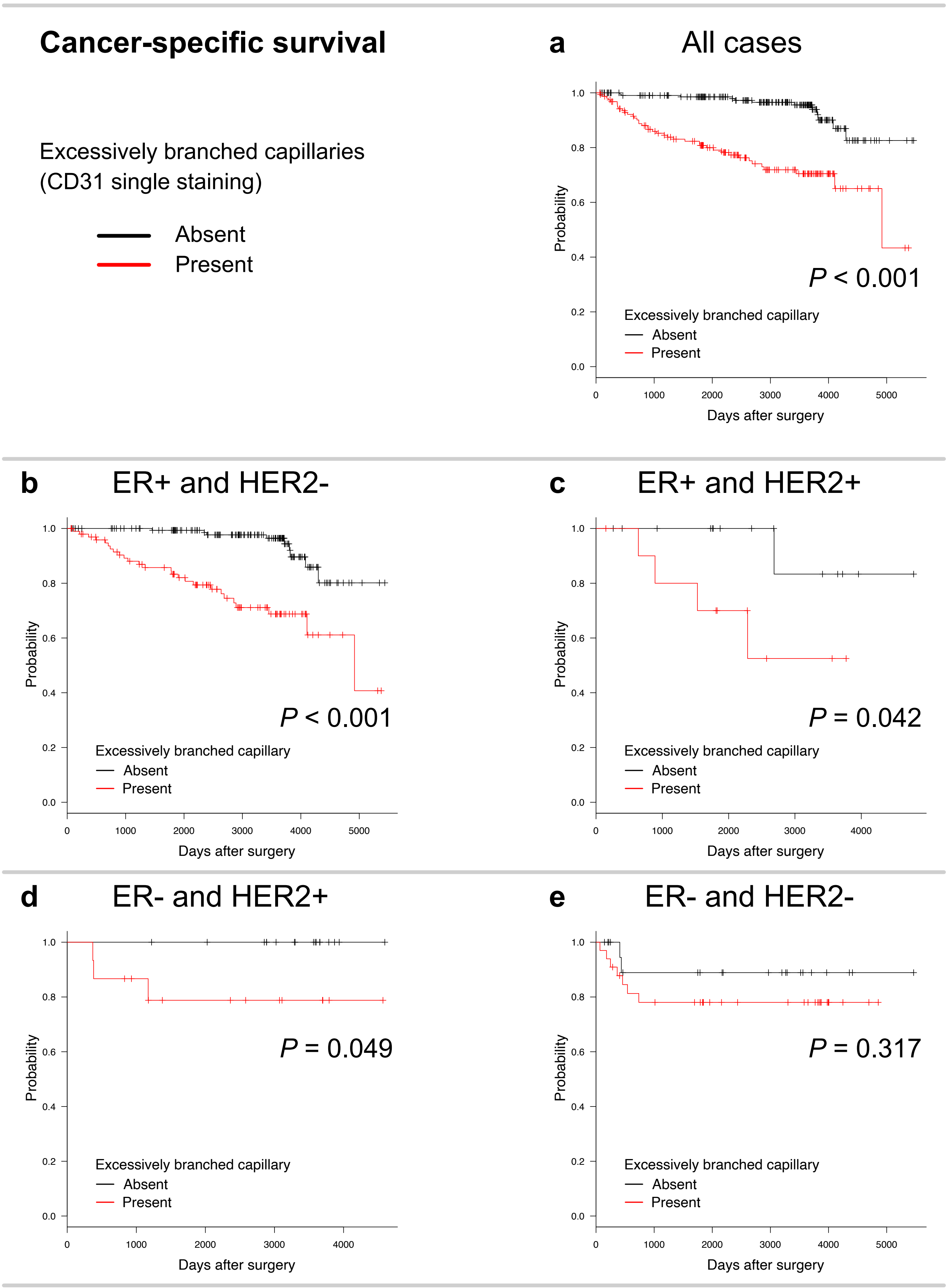

Supplementary Fig S3 Kaplan Meier survival curves of patients with and without excessively branched capillaries for cancer-specific survival

a: All cases (n = 376)

b: ER-positive and HER2-negative group (n = 263)

c: ER-positive and HER2-positive group (n = 26)

d: ER-negative and HER2-positve group (n = 32)

e: ER-negative and HER2-negative group (n = 55)

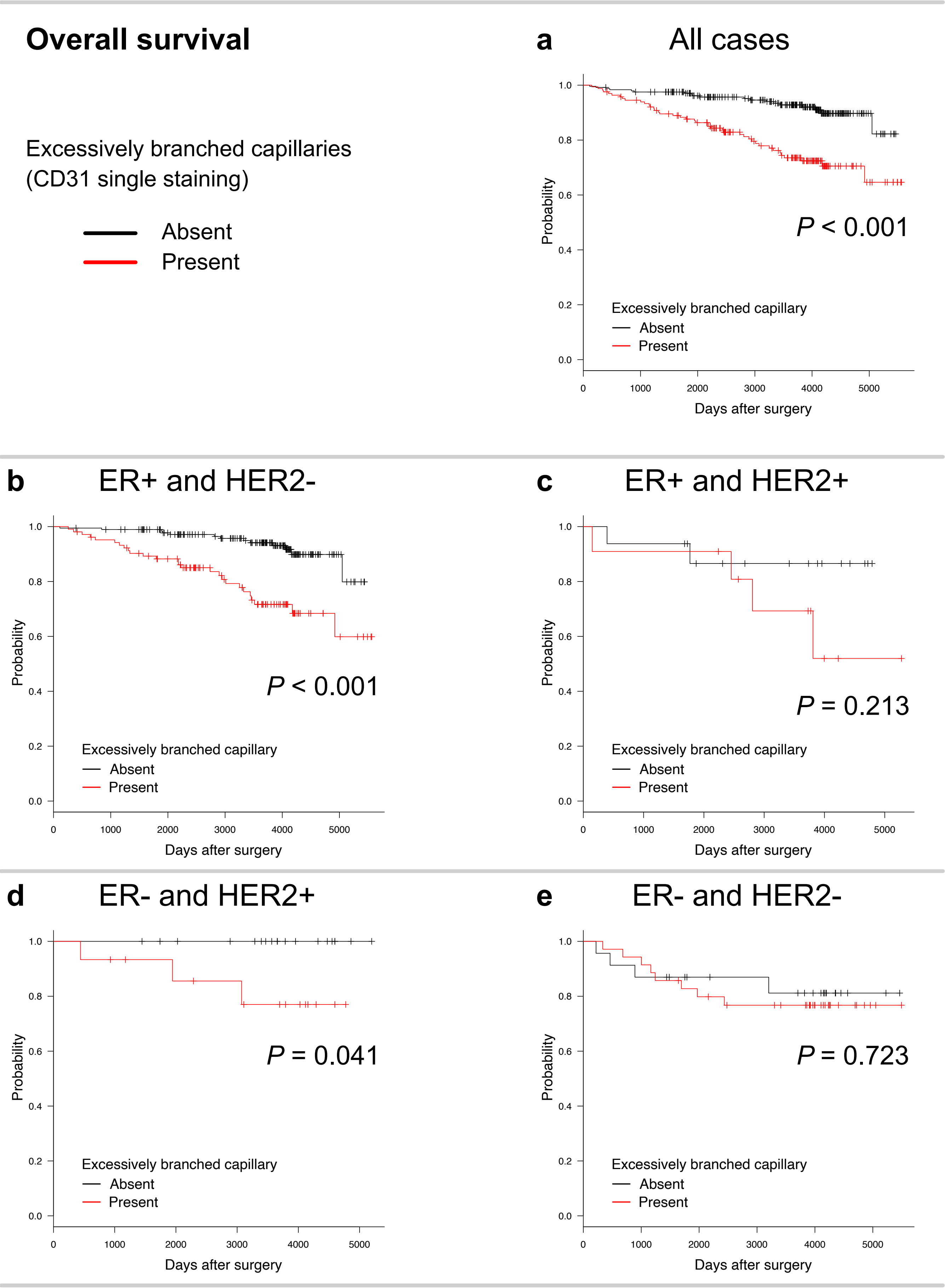

Supplementary Fig S4 Kaplan Meier survival curves of patients with and without excessively branched capillaries for overall survival

a: All cases (n = 407)

b: ER-positive and HER2-negative group (n = 288)

c: ER-positive and HER2-positive group (n = 27)

d: ER-negative and HER2-positve group (n = 34)

e: ER-negative and HER2-negative group (n = 58)

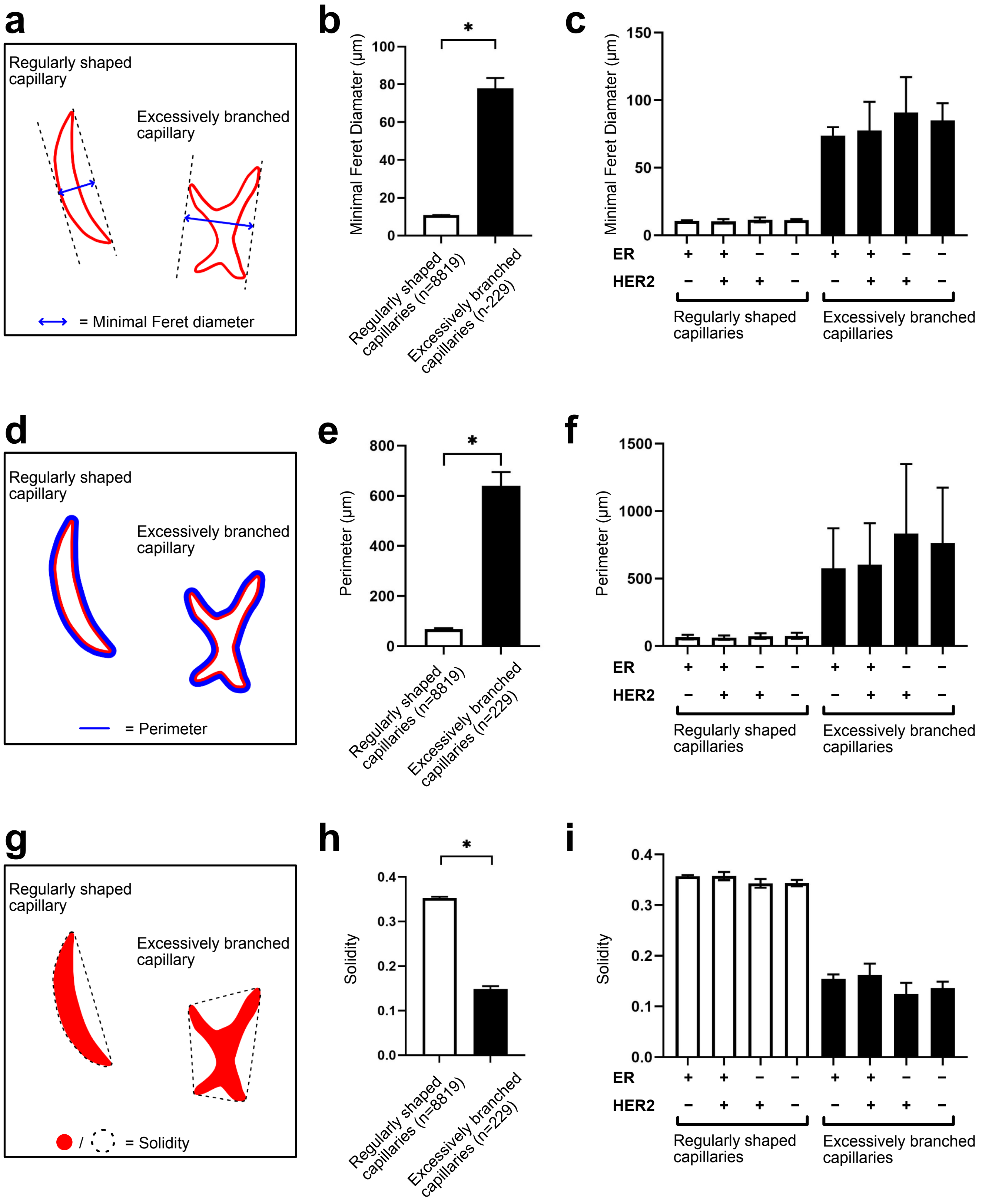

Supplementary Figure S5 Morphometric comparison of regularly shaped and excessively branched capillaries in invasive breast cancer

a,b,c: Size of blood vessels indicated by minimal Feret diameter. Minimal Feret diameter (length of blue arrow) is the distance between two parallel tangents (dotted lines) of the analyzed object (red) **(a)**. Minimal Feret diameter of all capillaries included in representative images for all cases with excessively branched capillaries **(b)**. Minimal Feret diameter displayed for each four subtypes **(c)**.
d,e,f: Size of blood vessels indicated by perimeter. Perimeter is the length of outline (blue) of the object (red)**(d)**. Perimeter of all capillaries for all cases **(e)** and for each four subtypes **(f)**.
g, h, i: Complexity of blood vessels indicated by solidity. Solidity is vascular area (red) divided by convex hull area (dotted line)**(g)**. Solidity of all capillaries for all cases **(h)** and for each four subtypes **(i)**. *$p<0.05$
