# Supplementary material for: The specific shapes of capillaries are associated with worse prognosis in patients with invasive breast cancer
Source: Pathol Int. 2024 May 31;74(7):394–407. doi: 10.1111/pin.13442 (PMC11551825; doi:10.1111/pin.13442)
Supplement: Supplementary file 3 — Supporting information. [file PIN-74-394-s003.pdf]

Supplementary Table S1. The clinicopathological characteristics of the patients with invasive breast cancer

Baseline patient and tumor characteristics		n	%
**Total**		411	100
**Age**	≤49	135	32.85
	≥50	276	67.15
**Tumor size**	≤2cm	298	72.51
	>2cm	113	27.49
**Lymph node metastasis**	Negative	280	68.13
	Positive	117	28.47
	Not accessible	14	3.41
**Histological grade**	Grade 1-2	268	65.21
	Grade 3	143	34.79
**Estrogen receptor**	Negative	93	22.63
	Positive	318	77.37
**Progesterone receptor**	Negative	128	31.14
	Positive	283	68.86
**HER2 overexpression**	Negative	350	85.16
	Positive	61	14.84
**Ki-67 index**	≤20%	231	56.2
	>20%	180	43.8
**Subtype**	ER+ and HER2-	291	70.8
	ER+ and HER2+	27	6.57
	ER- and HER2+	34	8.27
	ER- and HER2-	59	14.36
**Histologic type**	No special type (NST)	354	86.1
	Invasive lobular carcinoma	19	4.6
	Mucinous carcinoma	15	3.6
	Invasive micropapillary carcinoma	6	1.5
	Other special types[†]	17	4.1
**Lymphatic vessel invasion**	Negative	299	72.75
	Positive	112	27.25
**Blood vessel invasion**	Negative	218	53.04
	Positive	193	46.96
**Fibrotic focus**	Negative	281	68.37
	Positive	130	31.63
**Outcome**	Disease progression[‡]	52	12.65
	Death of any cause[§]	60	14.60

[†] Other special type: Tubular, Metaplastic, Apocrine, Matrix producing, Squamous, Spindle, Adenoid cystic, Cribriform, Combine

[‡] 35 cases: no information, Follow-up period: Median 2941 days (range 67-5464)

[§] 4 cases: no information, Follow-up period: Median 3701 days (range 111-5564)

Supplementary Table S2. Antibody and Staining Conditions

Antibody	Clone	Dilution	Pretreatment	Manufacturer
Collagen IV	CIV22	1:500	EDTA (pH 8.0), proteinase K	Cell Marque, Rocklin, CA
CD31 †	EP78	1:100	EDTA (pH 8.0)	Cell Marque, Rocklin, CA
CD31‡	EPR3094	1:500	EDTA (pH 8.0)	Abcam, Cambridge, UK
Podoplanin (D2-40)	D2-40	1:50	EDTA (pH 8.0)	Dako, Carpinteria, CA
GATA 3	L50-823	1:250	EDTA (pH 8.0)	Cell Marque, Rocklin, CA

† Used for single (CD31) and double (CD31 and CollagenIV) staining on thin sections.
‡ Used for thick sections.

Supplementary Table S3. CD31 and collagen IV double staining procedure

Step	Process
1	Deparaffin slides in xylene, followed by 100%, 90% and 80% ethanol
2	Rinse slides with water
3	Enclose sections in a pressure cooker with 0.5 M EDTA (pH 8) and microwave (700W) for 20 minutes
4	Cool down at room temperature
5	Primary antibody incubation for 90 minutes [†]
6	Secondary antibody[‡] incubation for 60 minutes [†]
7	Color detection with ALP[§]
8	Incubate slides in 0.004% proteinase K[¶] for 3 minutes [†]
9	Incubate slides in 10% H_2O_2 for 10 minutes [†]
10	Primary antibody incubation for 90 minutes[†]
11	Secondary antibody incubation for 60 minutes [†]
12	Histochemical reaction with DAB[‖]
13	Counterstaining with hematoxylin
14	Dry slides on a hot plate (50 °C) for 20 minutes
15	Mount sections by a conventional method

All the process was performed at room temperature.

[†] Followed by a rinse with Tris-buffered saline with Tween 20, three times.

[‡] Simplestain MAX-PO, Nichirei, Tokyo, Japan

[§] ImmPRESS Alkaline Phosphatase Polymer Reagent, Vector, Burlingame, CA

[¶] Cat.No.03115887001, Roche, Mennheim, Germany

[‖] Liquid DAB+ Substrate Chromogen system, Dako, Santa Clara, CA

Supplementary Table S4. CD31 single staining procedure

Step	Process
1	Deparaffin slides in xylene, followed by 100%, 90% and 80% ethanol
2	Rinse slides with water
3	Enclose sections in a pressure cooker with 0.5 M EDTA (pH 8) and microwave (700W) for 20 minutes
4	Cool down at room temperature
5	Incubate slides in 10% H_2O_2 for 10 minutes[†]
6	Primary antibody incubation for 90 minutes[†]
7	Secondary antibody[‡] incubation for 60 minutes[†]
8	Histochemical reaction with DAB[§]
9	Counterstaining with hematoxylin
10	Mount sections by a conventional method

All the process was performed in room temperature.
[†] Followed by a rinse with Tris-buffered saline with Tween 20, three times
[‡] Simplestain MAX-PO, Nichirei, Tokyo, Japan
[§] Liquid DAB+ Substrate Chromogen system, Dako, Santa Clara, CA

Supplementary Table S5. Staining procedure of thick FFPE sections (50μm)

Step	Process
1	Deparaffin sections in overnight xylene followed by 100%, 90% and 80% ethanol
2	Rinse sections with water
3	Enclose sections in autoclave machine with 0.5 M EDTA buffer (pH 8) and heat at 105°C for 20 minutes
4	Cool down at room temperature
5	Incubate slides in serum free bocking buffer for 60 minutes
6	Primary antibody (CD31 and GATA3) incubation at 4℃ overnight[†]
7	Secondary antibody[‡] incubation at 4℃overnight[†]
8	Place sections on glasses and mount sections by aqueous mounting medium[§]

[†] Followed by a rinse with Tris-buffered saline with Tween 20, three times
[‡] Secondary antibodies: Alexa Fluor 488 goat anti mouse IgG and Alexa Fluor 647 goat anti rabbit IgG (1:250), Invitrogen, Carlsbad, CA
[§]Aquatex mounting medium, Merck, Burlington, MA

Supplementary Table S6. Correlation of MVD and clinicopathological factors

	MVD (single staining)			MVD (double staining)		
	Low	High	P value	Low	High	P value
Parameter	n (%)	n (%)		n (%)	n (%)	
**Total**	173 (42.1)	238 (57.9)		165 (40.1)	246 (59.9)	
**Tumor size**			0.044*			0.144
≤2cm	116 (67.1)	182 (76.5)		113 (68.5)	185 (75.2)	
>2cm	57 (32.9)	56 (23.5)		52 (31.5)	61 (24.8)	
**Lymph node metastasis**			0.374			0.501
Negative	113 (68.1)	167 (72.3)		107 (68.6)	173 (71.8)	
Positive	53 (31.9)	64 (27.7)		49 (31.4)	68 (28.2)	
**Histological grade**			0.173			0.461
Grade 1-2	106 (61.3)	162 (68.1)		104 (63.0)	164 (66.7)	
Grade 3	67 (38.7)	76 (31.9)		61 (37.0)	82 (33.3)	
**Estrogen receptor**			1.000			0.549
Negative	39 (22.5)	54 (22.7)		40 (24.2)	53 (21.5)	
Positive	134 (77.5)	184 (77.3)		125 (75.8)	193 (78.5)	
**Progesterone receptor**			1.000			0.828
Negative	54 (31.2)	74 (31.1)		50 (30.3)	78 (31.7)	
Positive	119 (68.8)	164 (68.9)		115 (69.7)	168 (68.3)	
**HER2 overexpression**			0.575			0.674
Negative	145 (83.8)	205 (86.1)		139 (84.2)	211 (85.8)	
Positive	28 (16.2)	33 (13.9)		26 (15.8)	35 (14.2)	
**Ki-67 index**			0.009*			0.265
≤20%	84 (48.6)	147 (61.8)		87 (52.7)	144 (58.5)	
>20%	89 (51.4)	91 (38.2)		78 (47.3)	102 (41.5)	
**Subtype**			0.758			0.833
ER+ and HER2-	120 (69.4)	171 (71.8)		113 (68.5)	178 (72.4)	
ER+ and HER2+	14 (8.1)	13 (5.5)		12 (7.3)	15 (6.1)	
ER- and HER2+	14 (8.1)	20 (8.4)		14 (8.5)	20 (8.1)	
ER- and HER2-	25 (14.5)	34 (14.3)		26 (15.8)	33 (13.4)	
**Lymphatic vessel invasion**			0.313			0.115
Negative	121 (69.9)	178 (74.8)		113 (68.5)	186 (75.6)	
Positive	52 (30.1)	60 (25.2)		52 (31.5)	60 (24.4)	
**Blood vessel invasion**			0.194			0.132
Negative	85 (49.1)	133 (55.9)		80 (48.5)	138 (56.1)	
Positive	88 (50.9)	105 (44.1)		85 (51.5)	108 (43.9)	
**Fibrotic focus**			0.592			0.001*
Negative	121 (69.9)	160 (67.2)		97 (58.8)	184 (74.8)	
Positive	52 (30.1)	78 (32.8)		68 (41.2)	62 (25.2)	

* P value < 0.05

Supplementary Table S7. Correlation of glomeruloid microvascular proliferation (GMP) and clinicopathological factors

	GMP (single staining)			GMP (double staining)		
Parameter	Absent n (%)	Present n (%)	P value	Absent n (%)	Present n (%)	P value
**Total**	366 (89.1)	45 (10.9)		320 (77.9)	91 (22.1)	
**Tumor size**			0.032*			0.002*
≤2cm	272 (74.3)	26 (57.8)		244 (76.2)	54 (59.3)	
>2cm	94 (25.7)	19 (42.2)		76 (23.8)	37 (40.7)	
**Lymph node metastasis**			0.861			0.595
Negative	248 (70.3)	32 (72.7)		221 (71.3)	59 (67.8)	
Positive	105 (29.7)	12 (27.3)		89 (28.7)	28 (32.2)	
**Histological grade**			< 0.001*			<0.001*
Grade 1-2	252 (68.9)	16 (35.6)		232 (72.5)	36 (39.6)	
Grade 3	114 (31.1)	29 (64.4)		88 (27.5)	55 (60.4)	
**Estrogen receptor**			< 0.001*			< 0.001*
Negative	70 (19.1)	23 (51.1)		50 (15.6)	43 (47.3)	
Positive	296 (80.9)	22 (48.9)		270 (84.4)	48 (52.7)	
**Progesterone receptor**			< 0.001*			< 0.001*
Negative	102 (27.9)	26 (57.8)		80 (25.0)	48 (52.7)	
Positive	264 (72.1)	19 (42.2)		240 (75.0)	43 (47.3)	
**HER2 overexpression**			1.000			0.245
Negative	311 (85.0)	39 (86.7)		276 (86.2)	74 (81.3)	
Positive	55 (15.0)	6 (13.3)		44 (13.8)	17 (18.7)	
**Ki-67 index**			< 0.001*			< 0.001*
≤20%	217 (59.3)	14 (31.1)		202 (63.1)	29 (31.9)	
>20%	149 (40.7)	31 (68.9)		118 (36.9)	62 (68.1)	
**Subtype**			< 0.001*			< 0.001*
ER+ and HER2-	269 (73.5)	22 (48.9)		246 (76.9)	45 (49.5)	
ER+ and HER2+	27 (7.4)	0 (0.0)		24 (7.5)	3 (3.3)	
ER- and HER2+	28 (7.7)	6 (13.3)		20 (6.2)	14 (15.4)	
ER- and HER2-	42 (11.5)	17 (37.8)		30 (9.4)	29 (31.9)	
**Lymphatic vessel invasion**						0.183
Negative	269 (73.5)	30 (66.7)	0.375	238 (74.4)	61 (67.0)	
Positive	97 (26.5)	15 (33.3)		82 (25.6)	30 (33.0)	
**Blood vessel invasion**			0.429			0.032*
Negative	197 (53.8)	21(46.7)		179 (55.9)	39 (42.9)	
Positive	169 (46.2)	24 (53.3)		141 (44.1)	52 (57.1)	
**Fibrotic focus**			0.004*			< 0.001*
Negative	259 (70.8)	22 (48.9)		234 (73.1)	47 (51.6)	
Positive	107 (29.2)	23 (51.1)		86 (26.9)	44 (48.4)	

*P value - < 0.05
